# Individual language experience modulates rapid formation of cortical memory circuits for novel words

**DOI:** 10.1038/srep30227

**Published:** 2016-07-22

**Authors:** Lilli Kimppa, Teija Kujala, Yury Shtyrov

**Affiliations:** 1 Cognitive Brain Research Unit, Cognitive Science, Institute of Behavioural Sciences, 00014 University of Helsinki, Finland; 2 Center of Functionally Integrative Neuroscience, Department of Clinical Medicine, Aarhus University, 8000 Aarhus C, Denmark; 3 Centre for Cognition and Decision Making, National Research University Higher School of Economics, Moscow, Russia

## Abstract

Mastering multiple languages is an increasingly important ability in the modern world; furthermore, multilingualism may affect human learning abilities. Here, we test how the brain’s capacity to rapidly form new representations for spoken words is affected by prior individual experience in non-native language acquisition. Formation of new word memory traces is reflected in a neurophysiological response increase during a short exposure to novel lexicon. Therefore, we recorded changes in electrophysiological responses to phonologically native and non-native novel word-forms during a perceptual learning session, in which novel stimuli were repetitively presented to healthy adults in either ignore or attend conditions. We found that larger number of previously acquired languages and earlier average age of acquisition (AoA) predicted greater response increase to novel non-native word-forms. This suggests that early and extensive language experience is associated with greater neural flexibility for acquiring novel words with unfamiliar phonology. Conversely, later AoA was associated with a stronger response increase for phonologically native novel word-forms, indicating better tuning of neural linguistic circuits to native phonology. The results suggest that individual language experience has a strong effect on the neural mechanisms of word learning, and that it interacts with the phonological familiarity of the novel lexicon.

In today’s global world, we face an increasing need to master more than one language. More and more people are exposed to and learn multiple non-native languages in formal or informal contexts. In addition to learning the native language (L1), second (L2) and further languages can be learnt concurrently, sometimes starting in early childhood, resulting in high language proficiency. Later in life, non-native languages can still be acquired at different ages of acquisition (AoA), resulting in variable proficiencies. The possible influence of such individual language acquisition experience on the brain’s capacity to learn is poorly understood. While the majority of second language learning studies have focussed on comparing the processing of L1 and L2 in bilinguals^1^,^2^,^3^, only few studies have investigated how the prior experience in learning one or more non-native languages (multilingualism) affects neural mechanisms underpinning new word acquisition.

Some behavioural evidence suggests a benefit of multilingualism in learning novel non-native words using a paired-associate task^4^, rote or keyword method^5^, as well as by pairing non-native words with their native translations and repeating them aloud^6^,^7^. Learning an artificial vocabulary over a 4-day training period, however, was found to be unaffected by previous L2 learning background^8^. Such inconclusive results may be due to several reasons: the novel words were trained for different periods of time (i.e. learning was measured after a short training or following longer training regimes with longer consolidation periods), subjects in the studies had variable proficiencies and AoAs in their multiple languages, and furthermore, the experimental tasks tapped into different domains of language processing. In many studies, the language learning histories of other languages than L1 and the particular L2 used in the experiment are not reported at all (e.g. Mårtensson *et al.*^9^; Hosoda *et al.*^10^).

Studies exploring the neural bases of L1 and L2 indicate the recruitment of neural networks that are largely shared between native and non-native language processing, but to a different degree based on proficiency and AoA^11^,^12^,^13^. Although the evidence is somewhat inconclusive and task-dependent, typically early AoA and high proficiency lead to more consistent overlapping brain activations between the non-native and native languages^12^,^14^,^15^,^16^. In contrast, later-learned languages and lower proficiency tend to elicit more variable activations^13^,^15^,^16^,^17^,^18^. Underpinning these differences in activation outcomes after language acquisition, different factors – the number of languages, AoA, proficiency, and the amount of exposure and use of the non-native languages – may all define the extent to which past language learning may influence acquisition of a novel non-native lexicon.

Such a variability in activation patterns could also explain individual differences in learning a new language. Successful learners of non-native word-forms demonstrated stronger learning-dependent functional connectivity between left and right supramarginal gyri than that found in poorer learners^19^. Greater activations in several regions over the left and right hemispheres were found to be associated with better word learning outcomes, studied in different modalities with variable training methods^8^,^20^. In high-proficient early bilinguals, poor behavioural perception of phonetic contrasts of their second language was associated with weak neural discrimination of both native and unfamiliar non-native contrasts but not of non-speech sounds, suggesting major individual differences specifically in the perceptual language as opposed to the general auditory capacity^21^. Stronger activation increases in the left posterior superior temporal sulcus and the left middle temporal gyrus in capable learners have, on the other hand, been linked to greater malleability of these language regions^20^. Thus, it stands to reason that previous experience in learning non-native languages may lead to increased neural plasticity, the degree of which varies across individuals. This suggestion, however, has not been directly tested to date.

Crucially, individual differences in language histories of multilinguals can be expected to influence the memory circuits for language (similarly as demonstrated in early vs. late bilinguals^1^,^3^). Furthermore, language experience likely affects the dynamics of the formation of such memory circuits attributed to the learning process, and should thus be taken into account when the acquisition of a previously unknown language is under investigation. Those word-learning studies that involved subjects with experience in more than two languages, however, have so far not distinguished between the different influences of various factors that could characterise multilingual subjects, such as AoA, individual proficiency, or the number of languages learnt^4^,^5^. Moreover, neural evidence of such putative effects of knowledge of more than two earlier acquired languages on the word-learning mechanisms in the brain is lacking.

Filling this gap was the goal of the current study. To fulfil it, however, a reliable neurophysiological measure of word learning is needed. It was recently shown that formation of memory representations for novel word-forms may occur rapidly and automatically and can be traced online by recording neurophysiological responses elicited by new lexical items in the process of their acquisition through repetitive exposure^22^,^23^,^24^,^25^. In these studies, neural responses to novel spoken words with native phonology exhibited a reliable enhancement following the divergence point of the spoken stimuli during 15–30 minute experimental sessions as opposed to relatively stable responses to already known words or unfamiliar non-speech sounds. This enhancement, underpinned by left fronto-temporal cortical sources, has been proposed to reflect new memory trace formation in the perisylvian cortical areas^22^,^23^,^25^ that appears to take place very rapidly in the process of exposure to new spoken stimuli. This neural learning effect appears specific to native phonology, demonstrates independence of attentional demands and, importantly, correlates with recognition and retrieval from memory after exposure^25^.

In the current study, we set out to exploit this online neurophysiological index of new word acquisition to explore individual differences in rapid neural memory-trace formation for phonologically native and non-native novel word-forms; we specifically focussed on investigating the putative effects of previous experience in learning languages on the build-up of new linguistic memory traces. To this end, we used a group of native Finnish speakers with variable backgrounds in non-native language learning. We collected their individual language-learning history as well as neurophysiological responses to new spoken words using electroencephalography (EEG). On one hand, cognitive control capacity is known to be enhanced in bilinguals^26^,^27^, and, on the other hand, the rapid neural memory-trace build up is a largely automatised process^25^. With this in mind, we explicitly modulated the subjects’ attention on the stimulus input and recorded EEG both in a passive listening condition and in an attentive stimulus-oriented task (ignore and attend conditions, respectively). We analysed relations between language history variables and the magnitude of electrophysiological response increase for novel word-forms using multiple linear regression. According to the accounts of increased neural plasticity for language in the multilingual brain^28^, we hypothesised that a more extensive non-native language learning history would benefit the establishment of memory traces for novel non-native items.

## Methods

### Subjects

Twenty-two healthy right-handed Finnish speakers volunteered to take part in the experiment (mean age = 24.09, s.d. = 3.94; 10 males). All subjects had normal hearing and no history of language, neurological, or psychiatric disorders. None were early onset bi/multilinguals, i.e. they came from monolingual families and had not attended day care or school with foreign language immersion. However, all had learnt several non-native languages, which is the standard in the Finnish school system. Subjects gave written informed consent and were compensated for their time. Ethical approval was obtained from the Ethics Review Committee for Human Sciences (University of Helsinki), and all procedures were carried out in accordance with the approved guidelines and regulations.

### Stimuli

We used three types of spoken disyllabic consonant-vowel (CVCV) stimuli as follows: (1) familiar native words, (2) phonologically native novel word-forms, and (3) novel word-forms comprising non-native phonology. Two sets of five different first syllables (ke, pe, po, pu, te, and ky, kä, pi, tä, pö, respectively) and second syllables (to, ti, ka, pu.ko, and ky, py, ki, ti, pö) were used. By recombining the same spoken syllables in different order through cross-splicing, the resulting disyllabic items formed either a known word or a novel word with matched acoustical-phonetic properties across the stimulus types. The non-native novel word-forms were constructed by cross-splicing morphed first syllables with the same second syllables used in the native items. By morphing two native syllables, we created five CV syllables with non-native phonological properties (pi|ta, pö|pu, te|pa, tö|pu, and pu|pä) that on average had the same physical properties as their native counterparts. The use of a morphing algorithm (TANDEM-STRAIGHT^29^; a sound signal decomposition technique that eliminates periodicity information, after which the interference-free spectrum, F0, and aperiodicity of the signals can be re-synthesised to form a novel sound signal) to create unfamiliar phonology instead of using phonemes of some non-native language ensured that none of the subjects had been exposed to these syllables prior to the experiment. Having identical second syllables for each stimulus type ensured matched physical properties across different stimulus categories (native known words, native novel word-forms, and non-native novel word-forms). This also enabled precise definition of the divergence point (i.e. the point in time when various stimuli diverged allowing identification of the lexical status at the same time) as the critical second syllable onset. Although the brain responses to native words were not relevant per se to the research question, presenting known word-forms among the novel items ensured the recognition of the native items to be possible only at the second syllable. All syllables were 145 ms in duration, separated with a 75 ms silent gap. Additional target stimuli used in the attend condition were created by prolonging the inter-syllable silent closure of the stimuli 70 ms; an equal number of targets were created from each stimulus category. The stimuli were then grouped into two different subsets for their use in attend and ignore conditions, and the use of these was counterbalanced across subjects and conditions. For a more comprehensive description of the stimulus preparation procedure, see Kimppa *et al.*^25^.

### Procedure

Subjects filled in a questionnaire concerning their history of learning non-native languages prior to the experiment. The subjects listed all the non-native languages they had learnt, the age of acquisition (AoA; onset of learning), and self-reported proficiency (Likert scale 1–5; 1 = basic, 2 = passable, 3 = good, 4 = commendable, 5 = excellent) for each language.

In the EEG experiment, auditory stimulation was binaurally delivered via headphones at 50 dB above individually-determined hearing threshold. The average stimulus onset asynchrony (SOA) was 850 ms, jittered to range from 800 to 900 ms. Each token was repeated 150 times pseudo-randomly and equiprobably in consecutive sub-series of fifteen trials to ascertain their balanced occurrence throughout the overall exposure. First, in the *ignore condition* the subjects were instructed to ignore the sound stimuli while watching a silent film. Thereafter, in the *attend condition* they were asked to pay close attention to another set of stimuli and ignore the film, and their task was to memorise the stimuli as well as press a button whenever an infrequent target stimulus occurred. The ignore condition always came first, in order to avoid possible carry-over effects of attention, and was followed by the attend condition. Subjects’ attention on the visual stimulation was monitored in both conditions with a questionnaire about the content of the film, which indicated their full compliance with the task.

### EEG recording and pre-processing

Electroencephalogram was continuously recorded with 64 active electrodes mounted in a cap, using the Biosemi EEG setup (Biosemi B.V., Amsterdam, Netherlands) at 512 Hz sampling rate in an acoustically and electrically shielded booth. Eye-movements were monitored by a vertical and horizontal electro-oculogram (EOG) placed below and laterally to the right eye.

The data were downsampled offline to 256 Hz, channels with noisy signal were interpolated and principal component analysis (PCA^30^) was used to remove artefacts produced by eye blinks. Data were then passband filtered between 0.5–45 Hz and epoched to 800-ms segments from stimulus onset; the period from stimulus onset to critical second syllable onset was used for baseline correction. Exclusion criteria for epochs included artefacts exceeding ±100 μV (EEG and EOG channels) and those containing responses to target stimuli. Reference was set to the mean of two separately placed mastoid electrodes. Averages for each stimulus type were calculated by combining epochs of the five tokens of known, native novel or non-native novel words separately. The first and last 25% of the trials in each condition were used to define change in the ERP dynamics between the early and late stages of the exposure (as in Shtyrov *et al.*^22^). BESA Research 6.0 (BESA Software GmbH, Münich, Germany) and Matlab R2012a (The MathWorks, Inc., Natick, MA, USA) software packages were used for the analyses.

### Statistical analysis

Event-related potentials from the early and late stages of exposure to the novel stimuli were obtained for both native and non-native novel word-forms in each condition separately. Mean amplitudes based on a priori-selected 20-ms time window around negative peaks of group waveforms at a ROI of Fz and FCz ~50 ms after the word divergence point (namely, the second syllable onset), previously shown to exhibit the learning-related enhancement for novel word-forms^25^, were used in the analysis.

The reported scores (proficiency level, age of acquisition, and time since AoA) were averaged across languages to form single variables of general language experience. This approach was chosen since it takes the entirety of learnt languages into account.

Putative connections of age and language to the neural response dynamics as well as relationships between the language variables serving as predictors were examined with Pearson’s correlation. Background variables that significantly correlated with the response change of any of the novel word-forms were entered into a multiple regression analysis in order to explore which of the language measures significantly predicted the neural response dynamics. A multiple linear regression model was calculated separately for each response change to novel word-forms (native and non-native) in both conditions (ignore and attend). Structures of the resulting regression models were compared between conditions and word-form types with Meng’s Z-test^31^ that compares correlated correlation coefficients in order to find out if there were significant differences in how well the model predicted the neural response changes to different word-form types in different conditions. Furthermore, to investigate if the regression coefficients of the predictor variables differed between models, we used the approach by Cohen^32^ in which the difference between the predictors’ regression weights is divided by the standard error of the difference and the resulting z-score is tested for significance. SPSS Statistics 21 software (IBM Corp., Armonk, NY, USA) was used for statistical analyses.

## Results

The ERP responses to novel spoken word-forms at ~50 ms after the stimulus divergence point, whose online increase has previously been shown to indicate neural representation build-up and behavioural word learning^25^ were a priori chosen as the key variable and an index of rapid neural plasticity for new word-forms. A considerable amount of variation in response development (i.e. change in the mean amplitude of the early negative peak over the exposure time) of non-native word-forms at the individual level was observed (mean response increase in both ignore and attend conditions = 0.24 μV (s.e.m = 0.30 and 0.34 μV, respectively)), whereas for novel native word-forms the variance was smaller and thus the negative-going response increase more prominent (mean change = 0.22 (s.e.m = 0.23) in ignore and 0.41 μV (s.e.m = 0.24) in attend condition). The F-test of the across-condition variances showed a significant difference (p = 0.026). This implies greater individual differences in learning-related dynamics for phonologically non-native than native input.

### Language experience

**Figure 1:**
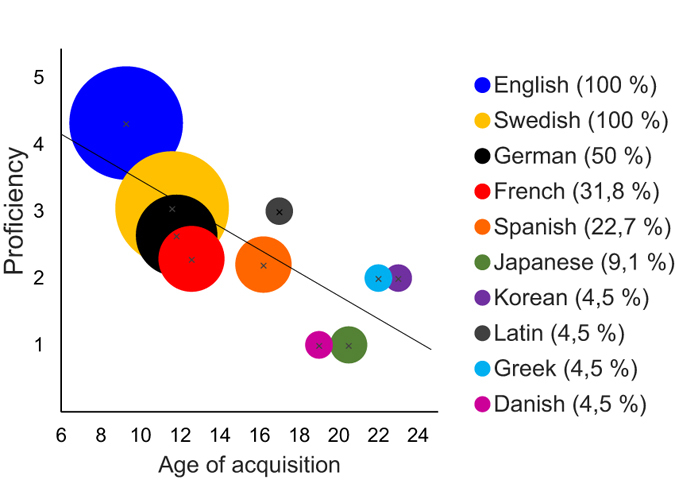
Average age of acquisition and proficiency of each reported non-native language. The size of the circle and number in the brackets denote the percentage of subjects who reported learning the language. Self-reported proficiency is shown on scale 1–5 (1 = basic, 5 = excellent). The age of acquisition correlated negatively with proficiency.

The data on language history are shown in Table 1 and Fig. 1. The majority, 86%, of the participants had English as the first non-native language learnt, the rest of them having Swedish. Two participants had an AoA earlier than school age for one language due to exposure to it in their neighbourhood. The reported proficiencies and AoAs showed a significant negative correlation (r = −0.745, p = 0.013) whereby the earlier a language had been acquired, the higher its proficiency was (Fig. 1).

There were no significant correlations between the subjects’ current age and the neural changes (all p-values > 0.11). Thus, age was left out from further analyses. The number of learnt languages and the average AoA showed significant simple correlations with at least one of the ERP changes for novel word-forms. These variables were therefore selected for the multiple regression to predict novel word-learning related neural changes.

### Regressions of language experience and neural dynamics

**Figure 2:**
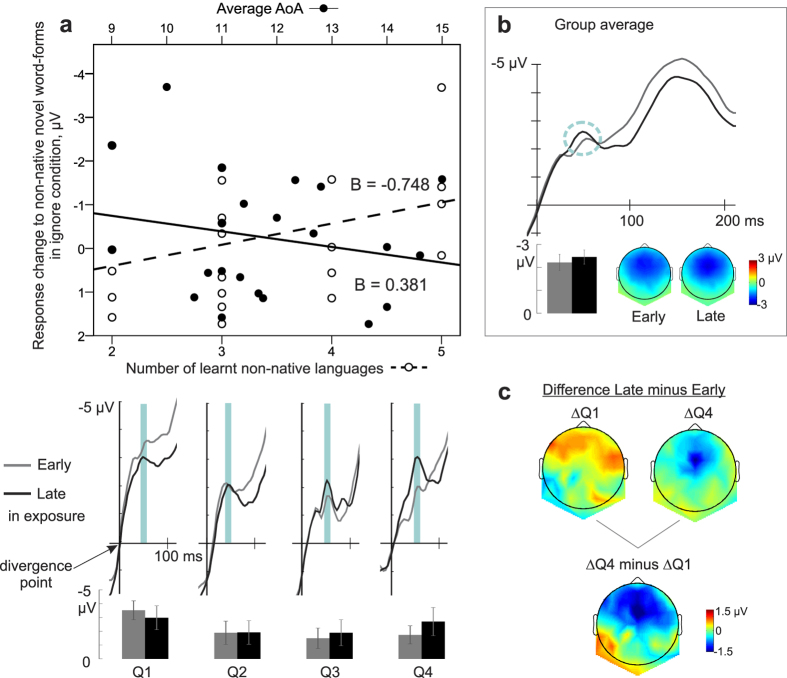
The influence of the number together with the average AoA of learnt non-native languages on the rapid response modulation of novel words with non-native phonology in the ignore condition. (**a**) The two predictors of the multiple regression model, i.e. the number of learnt non-native languages (open dots and dashed line) and the average AoA (solid dots and line), significantly predicted the response increase to the non-native novel word-forms in the ignore condition (top) registered at ~50 ms after the word divergence point (indicated by the y-axis). The more learnt languages and the earlier average AoA, the larger the response increase between early and late stages in exposure to non-native novel word-forms was. Waveforms and histograms representing average response early (grey) and late (black) during the exposure to the non-native novel word stimuli for each subgroup by quartile (Q1–Q4) are shown to illustrate the differences in response development (bottom); the subjects were subgrouped such that those with the lowest number of learnt languages with highest average AoA belong to the first quartile subgroup (Q1) and those with the highest number of learnt languages together with the earliest average AoA are in the subgroup of the fourth quartile (Q4). Error bars denote standard errors of mean. (**b**) The group average of the response to the non-native word-forms and the corresponding scalp topographies at early (grey) and late (black) stages of exposure. Waveforms are from a sensor ROI combining Fz and FCz where the responses were strongest at the ~50 ms, i.e. the latency at which the learning-related neural response increase for novel native word-forms at the group level was elicited (denoted by the circle). The consecutive peak at ~150 ms did not reflect rapid learning effects^25^, and was not analysed further here. (**c**) Scalp distributions of the difference between early and late stages of exposures for the first and last quartiles and the subtraction of their distributions.

**Figure 3:**
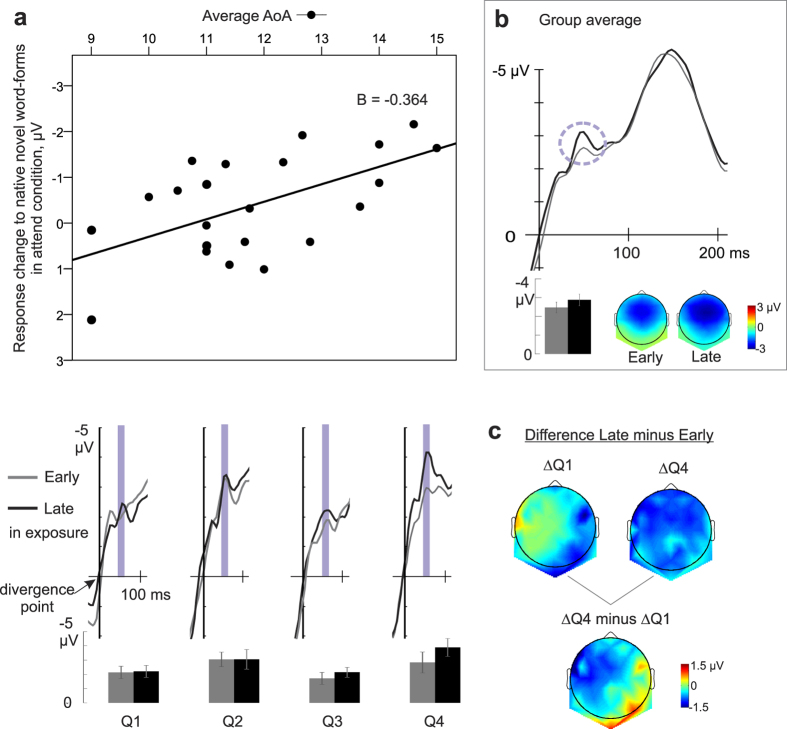
The effect of average AoA on the neural dynamics of native novel word-forms in the attend condition. (**a**) Individual magnitude of response change at ~50 ms to native novel word-forms in the attend condition was predicted by the average AoA of non-native languages (top). The later the AoA, the greater the response increase. Waveforms and histograms of response dynamics of the native novel word-forms in each quartile subgroup (Q1–Q4) of average AoA are presented below the regression plot; subjects with the lowest average AoA in Q1 and those with the highest average AoA in Q4. Error bars denote s.e.m. (**b**) The group-level response curve and scalp distribution early and late in exposure at the learning-related peak ~50 ms (denoted by the circle) post divergence point where the response increase was significant^25^. The later peak at 150 ms did not demonstrate learning-related dynamics^25^. (**c**) Scalp distributions of the difference between early and late stages of exposure for the first and last quartiles and the subtraction of their distributions.

The multiple linear regressions revealed significant associations between two background variables of language learning and ERP response dynamics. Low level of multicollinearity was present for the predictor variables (tolerance = 0.813, VIF = 1.231). The regression model for response increase to non-native novel word-forms in the ignore condition was statistically significant (F(2, 19) = 3.866, p = 0.039, R^2^ = 0.289), with significant regression coefficients for the number of languages (B = −0.748, p = 0.019) and the average AoA (B = 0.381,p = 0.05). The trend was similar in the attend condition (F(2, 19) = 3.053, p = 0.071, R^2^ = 0.243) in which the number of learnt languages had a significant regression coefficient (B = −0.838, p = 0.024) whereas that of the average AoA did not reach significance (B = 0.268, p = 0.224). In other words, the more non-native languages learnt with an earlier average starting age, the more the negative response to novel *non-native* word-forms increased (Fig. 2). In addition, the response increase for novel native word-forms in the attend condition was significantly predicted by our model (F(2, 19) = 4.789, p = 0.021, R^2^ = 0.335), with a significant coefficient for the average AoA (B = −0.364, p = 0.016) but not for the number of learnt languages (B = −0.068, p = 0.762). That is, the later the non-native languages were learnt on average, the more the neural response to novel *native* word-forms increased (Fig. 3). The regression model for novel native response change in the ignore condition was not significant (F(2, 19) = 2.466, p = 0.112). Noticeably, there were no significant correlations between the predictors and the original responses to novel word-forms at the early or late stages of exposure (all p-values > 0.054), excluding the possibility that the significant regressions obtained above were driven by individual differences in basic responses.

The correlation coefficients of the models predicting response changes to novel native and non-native word-forms were compared with Meng’s Z test. No such differences were present either between the ignore and attend conditions for the non-native (z = 0.735, p = 0.463) and native word-forms (z = −0.514, p = 0.607), or between the native and non-native word-forms in the ignore (z = −1.159, p = 0.246) and attend (z = 0.354, p = 0.723) conditions. Although, strictly speaking, this lack of difference per se does not indicate that the models explained identical amount of variance, the result generally suggests that the structure of the model was reasonable for each case. Further scrutiny of the predictors revealed significant differences only when comparing their weights for response changes to native and non-native word-forms in the attend condition (z = −2.514, p = 0.032 for number of learnt languages, and z = −2.508, p = 0.012 for average AoA). This confirms that in the models, the associations between language variables and response changes to non-native vs. native word-forms in the attend conditions were dissimilar, i.e. the predictors showed opposite directions of effects, especially in the case of average AoA for which the models provided significant coefficients for the non-native as well as for native word-form dynamics. Comparisons of the weights for different word types in the ignore condition, and for each word type across conditions were not significant (|z| < 1.301, p-values > 0.188).

## Discussion

The current study aimed at determining whether previous experience in language learning may influence rapid learning-related neural dynamics that has recently been suggested to reflect the automatic build-up of neocortical memory traces for novel words^22^,^23^,^24^,^25^ in the adult brain. To this end, we recorded brain’s electrophysiological responses during repetitive exposure to novel spoken items with native and non-native phonology, and obtained the subjects’ individual language acquisition background. Our subjects were native Finnish speakers who had learnt multiple languages at different levels of proficiency but were not early bilinguals (i.e. they had not acquired non-native languages in infancy or early childhood). We found that, first, the average AoA and the number of learnt languages predicted how much the response to non-native novel word-forms increased during passive exposure. Namely, earlier AoA with greater number of acquired languages was associated with a greater response increase. The predictive model in the attend condition approached but failed to reach significance, possibly indicating a greater role for these factors in automatic (rather than in controlled) word-form acquisition. Second, we found that the average AoA of non-native languages significantly influences rapid neural memory trace formation for novel native words, later onset predicting larger increase in the response to attended novel native words during the exposure. These results indicate that the brain’s readiness to develop new memory circuits for novel words of familiar or unfamiliar phonology is affected by the availability and extent of pre-existing networks for different languages.

The learning-related dynamics observed for novel words with non-native phonology was linked with the number of previously learnt languages and their average AoA. Learning of non-native languages often requires learning novel phonetic, phonological, or even tonal contrasts. The non-native word-forms used in the current study were constructed by cross-splicing together an unfamiliar non-native consonant-vowel syllable with a final syllable with familiar phonology. The final syllables were identical in both native and non-native word types. Thus the different magnitude of the neural response increase for these items (as opposed to the phonologically fully native ones) could only be linked to the processing of the non-native first syllables. It has been suggested that neural plasticity behind learning novel contrasts relies on the successful acquisition of auditory-articulatory (perception-action) mappings of speech^33^. Their successful acquisition, however, is variable. Individual differences were found in learning to identify novel contrasts: individuals with better training-induced performance were shown to recruit the frontal speech areas to a greater extent in addition to showing stronger activity to native contrasts than poorer learners^34^. Moreover, Díaz *et al.*^21^ demonstrated that the individual variability in the ability to identify and discriminate contrasts was speech-specific with no differences in basic auditory discrimination found between good and poor perceivers of L2 and unfamiliar contrasts. Finally, it was shown that fully-fledged left-lateralised word memory circuits may for their development require articulatory practice of the new phonology, benefiting from temporo-frontal perception-action circuitry^35^. Together, these results suggest that it is the speech-specific perception-action neural machinery that enables learning words with novel phonology. However, future studies also need to address the significance of similarity between the novel and previously learnt phonologies in how it might influence the efficiency of new language learning.

Learning of multiple languages requires familiarisation with novel speech sounds not included in the native language. Such familiarisation with novel phonemes that has occurred during learning more rather than fewer languages might engender a positive relationship between the number of learnt non-native languages and the magnitude of rapid learning response increase for novel non-native input in the current study. Due to the language experience, the underlying circuitry may have become more flexible to obtain new phonemes. Our results thus suggest that the plastic properties of the word learning network are defined by the pre-existing experience in language learning. Moreover, we found that the rapid memory trace formation for non-native words benefits from early AoA of the learnt languages, ranging from childhood to early adolescence (9–15 years in the current study). These results support behavioural evidence gained with more explicit word learning paradigms regarding the advantage of speaking multiple languages for word learning in an unknown language^4^,^5^,^6^,^7^, and provide novel information about factors predicting individual differences in neural encoding of novel word-forms.

The relationship between the number and average AoA of the learnt languages and the response increase to non-native languages was significant in the condition where the speech input was ignored but also trended similarly when the spoken stimuli were attended to and actively memorised. This suggests that attention was not crucial for mediating the effect of language learning experience on neural changes; rather, it seems that the plastic mechanisms that have enhanced through previous language experience may interact automatically with the neural learning dynamics for phonologically novel word-forms. To explain the benefits of multilingualism in the initial encoding of novel words, previous studies have implied greater flexibility and resistance to inhibit interference from L1 in early high proficient bilinguals^17^,^36^. This would indicate enhanced cognitive control in individuals speaking multiple languages compared to monolinguals. Beyond superior ability of inhibitory control in language learning^37^, this suggestion has been backed up by results of bilingual young adults outperforming monolinguals in tasks with high cognitive control demands whereas a more general advantage of bilingualism in executive control is only demonstrated by children and older adults^38^,^39^. In the early stages of non-native word learning, more localised articulatory control mechanisms were activated in bilinguals than monolinguals suggesting that extensive experience in managing multiple languages influences the recruitment of the cognitive control network^40^. Some studies reported better phonological short-term memory in multilinguals (e.g. Papagno and Vallar^4^) compared to monolinguals, but it was not found to be the defining factor of the bilingual advantage in word learning when the memory spans were matched between groups^41^. The results of the current study indicate that rather than requiring inhibitory control of the acquired native or non-native languages^36^,^40^, indivudual language experience modulates the rapid neural dynamics for novel non-native words automatically and independently of controlled attention. This is supported by the results from the ignore condition where no active task focussed on the spoken stimuli was employed and, furthermore, the attention was distracted from them to the visual input.

While unambiguously documenting functional neurophysiological changes and their linkage with individual language experience, the current study cannot explain whether the registered individual differences were purely functional in nature or whether they are underpinned by any structural variability in the brain’s neuroanatomy. Previous studies documented changes in grey and white matter organisation in several language areas in both left and right hemispheres after acquiring two^9^,^10^,^42^,^43^ or more languages^44^,^45^,^46^. For instance, white matter tracts showed progressive reorganisation in both hemispheres across 9 months of language education^43^. Furthermore, after intensive training of vocabulary over months, the resulting competence in the learnt language was positively correlated with the increased grey matter volumes^9^,^10^ and density^47^. Mechelli *et al.*^42^ found a common site of grey matter density increase in early and late bilinguals compared to monolinguals in the left inferior parietal cortex; the degree of the increase was modulated by the attained proficiency and AoA of the L2. Specifically related to our findings, varying AoA^48^ and number of learnt languages^44^ were related to distinguishable structural properties of the language network. As our subjects had learnt several non-native languages for multiple years, we may hypothesise that the functional effects here could at least in part be underpinned by such structural phenomena. This, however, cannot be presently confirmed and future studies are necessary to investigate whether neuroanatomical factors may underpin the rapidly changing dynamics of functional electrophysiological responses during the online acquisition process and its experience-dependant individual variability.

Somewhat surprisingly, the effect of experience in non-native languages was not limited to learning non-native input during the experiment. In the attend condition, the learning-related dynamics of words with native phonology was influenced by the average acquisition age of non-native languages: The later the learning of non-native languages had started, the more responsive the brain was to establish memory-traces (reflected by the response increase) to words with native speech sounds. This may imply that the average AoA of non-native language learning affected the degree of “neural commitment”^49^ to the native language input. The formation of new memory circuits for words was thus biased to native language for its dominance in the early language experience. It is notable, however, that the average AoA in the current study ranged from 9–15 years, when foreign languages are typically introduced in the Finnish schooling system. Thus, the ‘early’ AoA here refers to ages traditionally considered ‘late’ for second language learning but our results suggest that even in the time frame of six years between childhood and puberty, the differences in AoA of the non-native language learning continue having an impact on further language learning. To the best of our knowledge, this is a novel finding, since, to date, there is no previous research on effects of late-acquired L2s on word learning in L1. The gravity of this finding needs to be evaluated from a cautious stance, since it was only found to be significant in the attend and not the ignore condition.

The overall variability in the response change for novel items over the course of exposure was greater for the non-native than native items. This suggests that the L1 was still highly dominant in the subjects; indeed, none of the subjects were early high-proficient multilinguals. Still, the effect of AoA on the novel native word response change implies that the learning of non-native languages in later as opposed to early childhood has an effect on how tuned the brain remains to the native language input. However, the association between average AoA and native word learning was found only in the attend condition. Focussing of attention on and active memorising of the words in the attend condition mediated the influence of the average AoA of non-native languages on the learning of novel native words. Indeed, the automatic memory trace formation for novel native material (in the ignore condition) seems to be unaffected or less influenced by the language learning experience. The interaction between AoA and both native and non-native word dynamics supports the evidence suggesting that the processing of L1 and non-native languages is to a large degree subserved by the same language network^3^.

The proficiency level in the learnt non-native language has been considered as a significant factor in determining the neurofunctional properties of the language^1^,^2^,^3^,^13^,^16^,^17^. The average proficiency in the non-native languages was not significantly associated with the rapid word learning-related dynamics. As a proficiency measure, we used the average of the self-reported proficiencies across all reported languages by a subject. This approach obscures proficiency differences between languages within individuals and the small sample size did not allow us to differentiate groups of across-language high or low proficiency. It should also be noted that in our study there was no correlation between the *average* proficiency and the number of learnt languages or *average* AoA. However, at the level of individual languages, a correlation usually seen as increasing proficiency with earlier AoA (e.g. Birdsong^50^), was present. Thus, the current data do not exclude the possibility that high vs. low proficiency levels across all acquired non-native languages could have a distinct effect on rapid neural memory-trace formation for novel words or word learning in general, which should be addressed in future studies.

In conclusion, this study determined the influence of language learning experience on novel word learning-related neural dynamics, which was previously shown to be indexed by an early electrophysiological response increase, elicited ~50 ms after the word disambiguation point and increasing in amplitude in the course of learning exposure^25^. We found two variables describing previous learning of non-native languages, namely the average age of acquisition and the number of learnt languages, to predict the magnitude of response increase for novel words during an intensive auditory exposure to them. Specifically, the earlier the acquisition of the learnt languages had started on average and the more non-native languages were learnt, the stronger the increase in response between the beginning and the end of exposure for novel non-native words was. In addition, AoA was positively associated with the response increase for novel native word-forms in the attend condition, whereby the later the non-native languages were acquired, the larger was the response increment for novel native words. These results demonstrate a significant role of earlier language experience in neural plasticity in general and in the rapid formation of memory circuits for novel words in particular. Critically, previous language learning not only influences how strongly the brain responds to novel non-native speech input but tentatively also to new words with native phonology. The benefit of acquiring multiple non-native languages in childhood thus extends to neural encoding of new words with unfamiliar phonology in adulthood, while, in contrast, earlier acquisition of non-native languages may be costly for the efficiency of rapid memory trace development for new native word-forms. These effects demonstrate how language learning experience modulates the degree and extent of neural plasticity for lexicon acquisition in adulthood.

## Additional Information

**How to cite this article**: Kimppa, L. *et al.* Individual language experience modulates rapid formation of cortical memory circuits for novel words. *Sci. Rep.* **6**, 30227; doi: 10.1038/srep30227 (2016).

## Figures and Tables

**Table 1:** Descriptive information of experience in non-native language learning.

	Mean (s.d.)	Range
Number of learnt non-native languages	3.32 (1.04)	2–5
Average age of acquisition (AoA)	11.84 (1.66)	9–14.6
Average time since AoA (years)	12.25 (3.36)	7.33–17.33
Average self-reported proficiency	3.13 (0.68)	2–3.67

Individual average across the number of learnt languages was calculated for each individual. Proficiency levels were estimated with a scale 1–5 (1 = basic, 5 = excellent). s.d. = standard deviation.
